# Soil and Foliar Applications of Silicon Mitigate Biotic Stress in Cape Gooseberry Plants Caused by Fusarium Vascular Wilt

**DOI:** 10.3390/biology15070536

**Published:** 2026-03-27

**Authors:** David Sebastián Chitiva-Sánchez, Ana María Pérez-Rincón, Cristhian Camilo Chávez-Arias, Hermann Restrepo-Díaz, Sandra Gómez-Caro

**Affiliations:** Universidad Nacional de Colombia, Sede Bogotá, Facultad de Ciencias Agrarias, Departamento de Agronomía, Carrera 30 No. 45-03, Bogotá 111321, Colombia; anmperezri@unal.edu.co (A.M.P.-R.); ccchaveza@unal.edu.co (C.C.C.-A.); hrestrepod@unal.edu.co (H.R.-D.)

**Keywords:** Andean fruit species, *Fusarium oxysporum*, disease management, *Physalis peruviana*, physiological responses, beneficial elements, soil-borne pathogens

## Abstract

Vascular wilt caused by *Fusarium oxysporum* f. sp. *physali* (Foph) severely limits cape gooseberry (*Physalis peruviana* L.) production in Andean regions, where management options are restricted and largely dependent on fungicides. Silicon (Si) has been proposed as a sustainable strategy to enhance tolerance to vascular pathogens; however, its role in the cape gooseberry–Foph pathosystem remains unknown. This study evaluated the effects of soil and foliar Si applications on disease development and physiological responses in cape gooseberry plants under greenhouse conditions. Three soil doses and three foliar doses were tested, including inoculated and non-inoculated controls without treatment. Si significantly reduced disease progression, decreasing the area under the disease progress curve (AUDPC) and disease severity index, with efficacy values of up to 69% in inoculated plants, particularly at 8 g kg^−1^ soil application. Si also reduced vascular browning and mitigated pathogen-induced physiological impairment by maintaining higher stomatal conductance, relative chlorophyll content, maximum quantum efficiency of photosystem II, and plant growth. These findings indicate that Si, especially when soil-applied, enhances physiological tolerance to Foph and represents a promising complementary tool for its integrated management.

## Simple Summary

Vascular wilt is a serious disease that affects cape gooseberry, an important fruit crop in the Andean region. The disease is caused by a soil-borne fungal pathogen and blocks the plant’s water-conducting tissues, leading to leaf yellowing, wilting, reduced growth, and even plant death. Current management mainly relies on chemical fungicides, which may increase production cost and have environmental impacts. This study evaluated whether silicon (Si), a naturally occurring mineral element, could help cape gooseberry plants to tolerate the disease. Si was applied either to the soil or sprayed on the leaves of the plants grown under greenhouse conditions. We found that plants receiving Si, especially through soil application, showed less disease symptoms and better growth compared to untreated infected plants. Si-treated plants also maintained healthier leaves and improved physiological performance under infection. These results indicate that Si can strengthen the plant’s natural ability to cope with the disease. Using Si as a complementary strategy could contribute to more sustainable and environmentally friendly management of vascular wilt in cape gooseberry production.

## 1. Introduction

Cape gooseberry (*Physalis peruviana* L.) is an important fruit crop due to its wide range of nutritional and medicinal properties [1]; consequently, its production has expanded across both tropical and subtropical countries [2]. Colombia is one of the main producers, with exports reaching 8541 tons of fresh fruit in 2022 [3,4]. In Colombia, three major diseases of economic importance have been reported in this crop: gray leaf spot (*Cercospora* spp.), dieback (*Phoma* sp.), and vascular wilt caused by *Fusarium oxysporum* f. sp. *physali* (Foph) [5]. The latter is the most limiting and causes the most severe damage. Its main impacts include reduced yield and fruit quality, as well as crop abandonment by farmers due to the persistence of the pathogen in the soil [2,6]. Management of this disease is particularly challenging because of the formation of resistant survival structures in the soil and the susceptibility of commercially available cultivars used by growers [7].

Silicon (Si) has been proposed as a sustainable strategy to enhance plant tolerance to vascular and other plant–pathogens [8]. Recent studies indicate that Si can exert beneficial effects in plant–pathogen interactions by activating defense-related enzymes, stimulating the production of antimicrobial compounds, and modulating signaling networks and genes associated with active plant defense [8,9,10]. In addition, Si has been reported to provide a physical barrier, as it accumulates in plant cell walls, conferring structural reinforcement against pathogen penetration [8,11,12]. The beneficial effects of Si have been observed in various plant species; however, most reports concern grasses such as rice, sugarcane, and certain Cyperaceae due to their high capacity to bioaccumulate this element [13,14,15]. Its positive effects have been described for foliar diseases caused by different pathogens in crops such as lettuce—*Bremia lactucae* [16], banana—*Mycosphaerella fijiensis* [17], coffee—*Hemileia vastatrix* [18], and wheat—*Blumeria graminis* [19]. For soil-borne diseases, beneficial effects of Si have been reported in oriental melon—*Fusarium* spp. [20], pepper—*Phytophthora capsici* [11], and coffee—*Meloidogyne exigua* [21]. In the case of vascular wilts caused by *F. oxysporum*, positive effects of Si have been reported in cucumber—*F. oxysporum* f. sp. *cucumerinum* [22], tomato—*F. oxysporum* f. sp. *radicis-lycopersici* [12], banana—*F. oxysporum* f. sp. *cubense* [23], and cotton—*F. oxysporum* f. sp. *vasinfectum* [24].

In recent years, the beneficial role of Si and its method of application, whether foliar or soil-based, in the prevention of diseases in dicotyledonous plants has gained increasing importance in tropical environments. Machado-López et al. [25] found that pre-harvest soil treatment with reduced potassium and soluble Si was most effective in controlling *Botrytis* during the post-harvest phase of rose cv. ‘Brighton’. Furthermore, Vargas-Rojas et al. [26] demonstrated that the application of Si, either to the soil or foliage, reduced leaf diseases, such as ascochyta blight, powdery mildew, and downy mildew, thereby decreasing the reliance on fungicides without affecting yield. Previous studies suggest that Si compounds can be incorporated into disease management strategies for dicot crops. However, to date, no studies have evaluated the effect of Si applications in the cape gooseberry–Foph pathosystem.

Considering the economic importance of this crop and the limiting nature of vascular wilt—largely due to the long-term persistence of the pathogen in soil through chlamydospore production [27] and its difficult control—the identification of alternative management strategies is urgently required. Given the promising reports of reducing vascular wilt severity caused by different *formae speciales* within the species *Fusarium oxysporum* following Si application, to evaluate its effect on vascular wilt caused by Foph in cape gooseberry is of significant interest. Therefore, the objective of this study was to assess the effect of different doses of Si applied either foliar or to the soil on the development of vascular wilt in cape gooseberry and on plant physiological performance. The findings suggest that Si is a promising alternative for managing vascular wilt in cape gooseberry crops. It not only helps to control the disease but also enhances plant behavior, making it a viable option for sustainable farming.

## 2. Materials and Methods

### 2.1. Plant Material and Growth Conditions

The experiment was conducted between February and June 2021 under greenhouse conditions at the Faculty of Agricultural Science, of the Universidad Nacional de Colombia, Bogotá campus (4°35′56″ N, 74°04′51″ W), with a mean temperature of 18 ± 8 °C, relative humidity ranging from 60 to 90%, and a natural photoperiod of 12 h.

Cape gooseberry (*P. peruviana*) seedlings of the “Colombia” ecotype, with three to four true leaves, were obtained from the Bio Systems Center Jorge Tadeo Lozano and transplanted into bags containing 1 kg of soil mixed with rice husk (3:1 *v*/*v*), previously autoclaved (121 °C, 15 PSI for 60 min). After transplanting, plants were irrigated every three days with 100 mL of a nutrient solution containing Nutri-ponic^®^ (Walco S. A., Funza, Colombia) at a dose of 5 mL L^−1^ of water during the experiment [28,29]. To confirm the absence of *F. oxysporum* in the plant material prior to inoculation, basal stem cross sections were evaluated on potato dextrose agar (PDA) (Oxoid^®^, Basingstoke, UK) for pathogen colony development 10 days after sowing (DAS) [30].

### 2.2. Plant Inoculation with Foph and Silicon Application

The Map5 strain of Foph, provided by the Biological Control Laboratory of the Colombian Corporation for Agricultural Research (Agrosavia, Mosquera, Colombia), was used as the pathogen source [7,31,32]. For inoculum production, 7-day-old mycelial plugs were transferred into 125 mL of potato dextrose broth (PDB) (Alpha Biosciences, Baltimore, MD, USA) in Erlenmeyer flasks and maintained under constant agitation for 15 days as described by Chávez-Arias et al. [29]. The inoculum volume and conidial density required for plant infection using the drench method were determined in preliminary assays. A concentration of 1 × 10^6^ conidia mL^−1^ and a volume of 80 mL kg^−1^ soil were used to achieve infection based on the protocol adjusted of Chávez-Arias et al. [29]. This inoculum density and suspension volume were used to evaluate Si treatments on vascular wilt development.

Two Si sources were used. For soil application, Silizeo-K (Agromil S. A. Ibagué, Colombia, 75% SiO_2_ and 3% K_2_O) was applied. Foliar applications were performed using MisilK 360 (Bioest S. A. S. Cota, Colombia, 8.2% K_2_O and 29.5% SiO_2_). The application methodology and use of these Si fertilizers were previously validated in tropical cropping systems [33,34]. Specifically, MisilK 360 was applied as a foliar spray, while Silizeo-K was applied at the base of the plant near the root zone at planting and subsequently covered with soil. For each Si source, three doses were evaluated: 2, 4, and 8 g kg^−1^ soil (soil application) and 0.5, 1, and 2 mL L^−1^ (foliar application). Two control treatments were included: inoculated plants without Si (Foph+) and non-inoculated plants without Si (Foph−). In total, eight treatments were evaluated: two Si sources (soil and foliar) at three doses each, plus two controls. The experiment was conducted twice over time.

### 2.3. Evaluation of Vascular Wilt Caused by Foph

Disease development was assessed weekly by visual inspection. Disease incidence (%) was calculated according to Zhang et al. [35]. Disease severity was evaluated using the severity scale described by Moreno-Velandia et al. [32], and the severity index was calculated following Townsend et al. [36].

The area under the disease progress curve (AUDPC) for each treatment was calculated according to Campbell et al. [37]. Treatment efficacy was calculated from AUDPC values using Abbott’s formula [38]:

(1) $$Efficacy\;\left(\%\right)=\frac{X-Y}{X}\;\times \;100$$

where *X* represents the AUDPC of the Foph+ treatment (inoculated without Si) and *Y* represents the AUDPC of each evaluated treatment.

**Figure A1:**
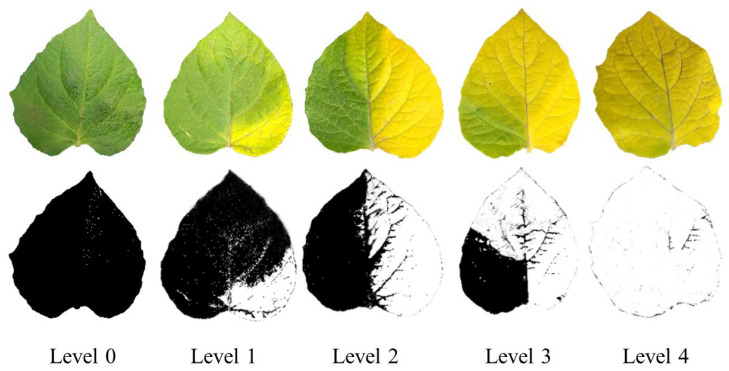
Diagrammatic scale to quantify the severity of vascular wilt caused by Foph on cape gooseberry (*Physalis peruviana* L.) leaves. 0 = healthy leaf with no chlorosis; 1 = leaf with chlorosis on 25% of the leaf blade; 2 = leaf with chlorosis on 50% of the leaf blade; 3 = leaf with chlorosis on 75% of the leaf blade; 4 = leaf with chlorosis on 100% of the leaf blade; over time, this leaf wilts and may remain attached to the stem or detach, causing defoliation.

Leaf chlorosis caused by vascular wilt was evaluated using a six-level diagrammatic scale developed in this study (Appendix A, Figure A1). The percentage of necrotic area at the stem base was quantified using cross stem and longitudinal sections analyzed with ImageJ software (V.1.53, National Institutes of Health, Bethesda, MD, USA). At 70 days after inoculation (DAI), the presence of Foph was confirmed by pathogen isolation from explants taken from stem bases on PDA (Oxoid^®^, Basingstoke, UK), incubated at 24 °C for 7 days, verifying typical colony growth and morphological characteristics of *F. oxysporum* [30,39].

### 2.4. Physiological and Growth Variables

Plant height was measured from 14 to 70 DAI at 7-day intervals. Relative growth rate (RGR) was calculated using Castro-Duque et al. [40]:

(2) $$RGR=\frac{Ln\;SL\;T2-Ln\;SL\;T1}{DS\;2-DS\;1}$$

where *SL T2* and *SL T1* are stem lengths at times 2 and 1, respectively, and *DS2* − *DS1* is the time interval in days.

At 63 and 70 DAI, leaf temperature (infrared thermometer, FLIR Systems Inc., Wilsonville, OR, USA), stomatal conductance (SC-1 porometer, Decagon Devices Inc., Pullman, WA, USA), relative chlorophyll content (RCC), and chlorophyll fluorescence parameters were measured using a MultispeQ V2 fluorometer (PhotosynQ, East Lansing, MI, USA). Chlorophyll fluorescence parameters included the maximum quantum efficiency of PSII (Fv’/Fm’), quantum efficiency of PSII under light conditions (φII), linear electron flow (LEF), photochemical quenching (qP), non-photochemical quenching (NPQt), uncontrolled non-photochemical dissipation (φNO), and controlled non-photochemical dissipation (φNPQ) were assessed. Measurements were performed on fully expanded upper-third leaves between 9:00 and 11:00 a.m. [29,41,42,43]. The relative tolerance index (RTI) was calculated from stomatal conductance data [44,45]:

(3) $$RTI=\left(\frac{g_{s}\;with\;Foph}{g_{s}\;without\;Foph}\right)\;\times \;100\;$$

At 70 DAI, leaf area (ImageJ V.1.53), fresh weight, dry weight, relative water content (RWC), and plant hydraulic conductance (K) were measured using a High-Pressure Flow Meter (HPFM, Dynamax Inc., Houston, TX, USA) [46]. RWC was calculated according to Equation (4) [47,48]:

(4) $$RWC=\left(\frac{FW-DW}{TW-DW}\right)\;\times \;100\;$$

where FW is fresh weight, TW is turgid weight (after 24 h distilled water at 4 °C), and DW is dry weight (after 48 h at 70 °C).

Finally, leaf proline concentration was quantified as a biochemical response using the acid ninhydrin method [49].

### 2.5. In Vitro Effect of Silicon on Mycelial Growth of Foph

The effect of both Si sources on Foph mycelial growth was evaluated using the amended medium technique [50,51,52]. PDA was amended with 0.5, 1, and 2 mL L^−1^ of MisilK 360 and 2, 4, and 8 g L^−1^ of Silizeo-K at 35–40 °C before pouring into Petri dishes. A 6-mm mycelial disk from a 7-day-old Foph colony was placed in the center of each plate. Plates were incubated at 22 ± 2 °C (Incucell, Brno-Zábrdovice, Czech Republic). Colony diameter was measured every three days for 15 DAS using a digital caliper (Steren, Mexico City, Mexico). The area under the mycelial growth curve (AUGMC) was calculated [37,53,54]. The experiment was repeated twice.

### 2.6. Experimental Design and Data Analysis

Both in vivo and in vitro experiments followed a completely randomized design (CRD). For the in vivo experiment, five plants per treatment were used as replicates for the physiological and growth variables assessment. Disease variables (severity index and AUDPC) were evaluated in groups of four plants per replicate and five replicates per treatment were used according to Chaves-Gómez et al. [55]. For the in vitro experiment, six Petri dishes per treatment were used as replicates.

Data were subjected to one-way analysis of variance (ANOVA), considering treatment as the main factor. When significant effects were detected (*p* ≤ 0.05), means were separated using Tukey’s honestly significant difference (HSD) test. All pairwise comparisons among treatments were performed. *p* values are reported according to standard conventions, and extremely small values are expressed as *p* < 0.001. Correlation analyses were performed between RTI and AUDPC, efficacy, RGR, total dry weight, RWC, K, Fv’/Fm’, and proline to determine the most effective treatments under Foph inoculation.

Data were analyzed using Statistix v9.0 (Analytical Software, Tallahassee, FL, USA). Principal component analysis was performed using InfoStat 2020 (analytical software, Universidad Nacional de Córdoba, Córdoba, Argentina) to visualize treatment relationships.

## 3. Results

### 3.1. Evaluation of Vascular Wilt Caused by Foph

**Figure 1:**
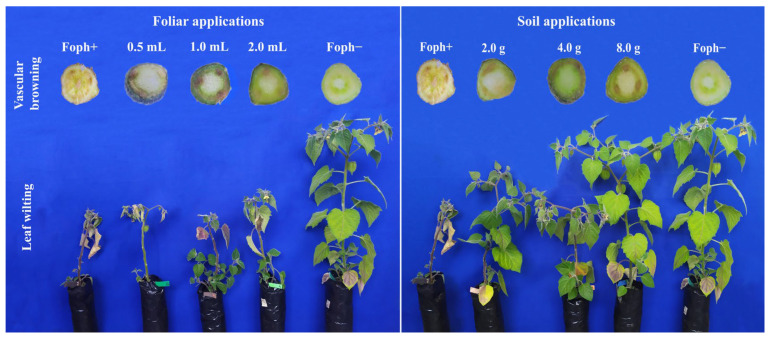
Cape gooseberry plants (*Physalis peruviana* L.) inoculated with Foph under different Si treatments showing typical symptoms of vascular wilt caused by Foph. Foph+: plants inoculated with Foph without Si; 0.5 mL: plants inoculated with Foph and foliar Si application at 0.5 mL L^−1^; 1.0 mL: plants inoculated with Foph and foliar Si at 1.0 mL L^−1^; 2.0 mL: plants inoculated with Foph and foliar Si at 2.0 mL L^−1^; 2.0 g: plants inoculated with Foph and soil-applied Si at 2 g kg^−1^ soil; 4.0 g: plants inoculated with Foph and soil-applied Si at 4 g kg^−1^ soil; 8.0 g: plants inoculated with Foph and soil-applied Si at 8 g kg^−1^ soil; Foph−: control plants without Foph and without Si.

The inoculated plants exhibited typical symptoms of the disease caused by Foph reported by Mendoza-Vargas et al. [56] and Cháves-Arias et al. [45]; however, disease severity varied according to the treatment (Figure 1). Plants subjected to foliar Si applications showed limited visual improvement, displaying symptoms similar to the inoculated control (Foph+), including pronounced leaf wilting and vascular browning. In contrast, soil-applied Si treatments resulted in a noticeable reduction in symptom severity, with a dose-dependent response. The highest soil dose (8.0 g) showed minimal vascular browning and healthy plant appearance comparable to the non-inoculated control (Foph−), while intermediate (4.0 g) and lower doses (2.0 g) exhibited moderate and slight improvements, respectively.

**Figure 2:**
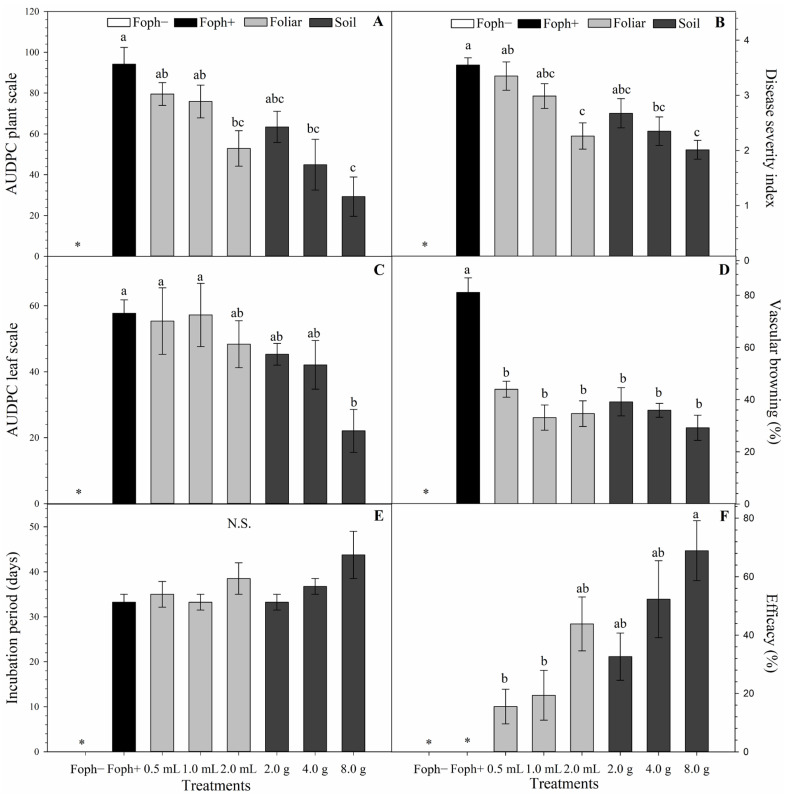
Development of vascular wilt in *Physalis peruviana* inoculated with Foph at 70 DAI under Si treatments. (**A**) AUDPC. (**B**) Disease severity index. (**C**) AUDPC calculated using the scale proposed in this study. (**D**) Vascular browning. (**E**) Disease incubation period. (**F**) Efficacy of Si treatments. Bars represent the standard error; values followed by the same letter are not statistically different according to Tukey’s test (*p* ≤ 0.05). NS, not significant. Asterisk (*) indicates plants with zero values for the evaluated variable.

Significant differences were found in AUDPC based on the disease severity scale of Moreno-Velandia et al. [32] (*p* = 0.0006), AUDPC using the scale proposed in this study (*p* = 0.0224), disease severity index (*p* = 0.0005), vascular browning (*p* < 0.0001), and treatment efficacy (*p* = 0.0069) (Figure 2A–D,F). The lowest AUDPC value (29.3), lowest vascular browning (29.2%), and highest efficacy (68.9%) were obtained with the highest soil-applied Si dose (8 g kg^−1^). Soil Si applications resulted in lower AUDPC values (29.3–63.4) and severity indices (2.0–2.7) compared to foliar applications, where AUDPC ranged from 52.9 to 79.5 and severity index from 2.3 to 3.3. Similar results were observed for AUDPC calculated using the proposed scale (Figure 2C), with soil-applied Si treatments ranging from 22.1 to 45.3, whereas foliar treatments ranged from 48.4 to 55.4.

For vascular browning (Figure 2D), soil treatments ranged from 29.2 to 39.2%, while foliar treatments ranged from 33.1 to 44.0%. The incubation period of vascular wilt (Figure 2E) ranged from 33.2 to 43.7 days for soil treatments and from 33.2 to 38.5 days for foliar treatments. In Foph+ control, vascular browning reached 81.1% and incubation period was 33.2 days. Although incubation period differences were not significant, a trend toward higher values was observed with Si application, particularly at higher doses.

Regarding treatment efficacy (Figure 2F), soil applications of 4 and 8 g and the highest foliar dose (2 mL) showed the highest efficacy values (52.3%, 68.9%, and 43.8%, respectively) compared to the Foph+ control.

**Figure A2:**
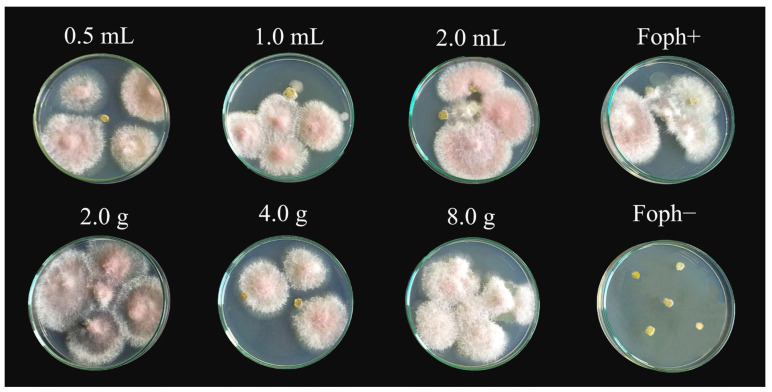
Confirmation of pathogen presence in plants under the different Si treatments evaluated. Typical Foph colonies were observed on PDA medium from explants of cape gooseberry plants subjected to foliar Si applications (0.5, 1, and 2 mL L^−1^ of water) and soil-applied Si (2.0, 4.0, and 8.0 g kg^−1^ of soil) after 30 days of incubation at 22 °C. Absence of colonies was observed in the control treatment without the pathogen (Foph−), whereas presence of colonies was confirmed in the treatment inoculated only with the pathogen (Foph+).

The presence of Foph in symptomatic plants was confirmed at 70 DAI through isolation on PDA medium (Appendix A, Figure A2). The obtained colonies matched the morphological descriptions for *F. oxysporum* [30,57].

### 3.2. Physiological and Growth Variables of Cape Gooseberry Plants

Significant differences among treatments were observed in RGR (*p* < 0.0001), leaf temperature at 63 and 70 DAI (*p* = 0.0012 and *p* < 0.0001), and stomatal conductance (*g_s_*) at 63 and 70 DAI (*p* < 0.0001). The highest RGR (0.0204 cm cm^−1^ d^−1^) was recorded in the 8 g soil-applied Si treatment, statistically similar to the non-inoculated control (Foph−). A similar pattern was observed for leaf temperature at 63 and 70 DAI in the 8 g soil treatment (23.3 °C and 20.6 °C), with no significant differences compared to Foph− (22.5 °C and 19.6 °C). For stomatal conductance, a general trend of higher *g_s_* values was observed in Si-treated plants. At 63 and 70 DAI, the 8 g soil treatment (269.1 and 341.1 mmol CO_2_ m^−2^ s^−1^) was statistically similar to Foph− (307.2 and 352.0 mmol CO_2_ m^−2^ s^−1^) (Table 1).

Regarding photosynthetic parameters, significant differences were observed in RCC at 63 and 70 DAI (*p* = 0.0018 and 0.0298), Fv’/Fm’ at 70 DAI (*p* = 0.0177), and LEF at 63 DAI (*p* = 0.0011). Other evaluated variables showed no statistical differences. RCC was highest in Foph− (48.2 SPAD at 63 and 70 DAI) and the 8 g soil treatment (46.8 and 43.4 SPAD at 63 and 70 DAI, respectively), without significant differences. The lowest values were observed in Foph+ without Si (31.2 and 27.2 SPAD). At 70 DAI, Fv’/Fm’ differed significantly among treatments. The highest value (0.67) was observed in the 8 g soil treatment, while the lowest values were found in Foph+ without Si (0.34) and 0.5 mL foliar Si (0.31) (Table 2).

**Figure 3:**
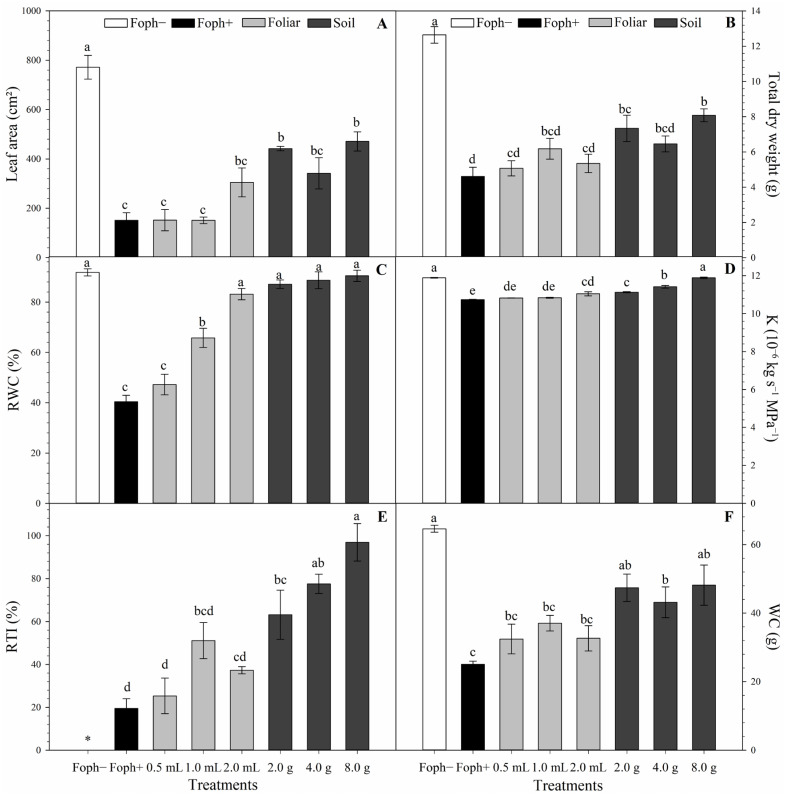
Effect of foliar and soil Si application on vascular wilt in cape gooseberry plants at 70 DAI: (**A**) leaf area (cm^2^); (**B**) plant dry weight (g); (**C**) RWC, %); (**D**) K (10^−6^ kg s^−1^ MPa^−1^); (**E**) RTI (%); and (**F**) WC (g). Bars represent the standard error, and letters indicate statistically significant differences according to Tukey’s test (*p* ≤ 0.05). Asterisk (*) indicates plants with zero values for the evaluated variable.

Leaf area, dry weight, RWC, K, RTI, and water content (WC) showed highly significant differences (*p* < 0.0001). Foph inoculation reduced leaf area and dry weight compared to Foph− (771.6 cm^2^ and 12.6 g). However, soil-applied Si increased these values compared to Foph+ and foliar treatments. The 8 g soil treatment showed significantly higher values (470.9 cm^2^ and 8.1 g). For RWC, K, RTI, and WC, soil applications—especially 8 g—showed the best performance, mitigating water stress caused by Foph and reaching values similar to Foph− (90.4%, 11.9 × 10^−6^ kg s^−1^ MPa^−1^, 96.9%, and 48.2 g, respectively) (Figure 3).

**Figure 4:**
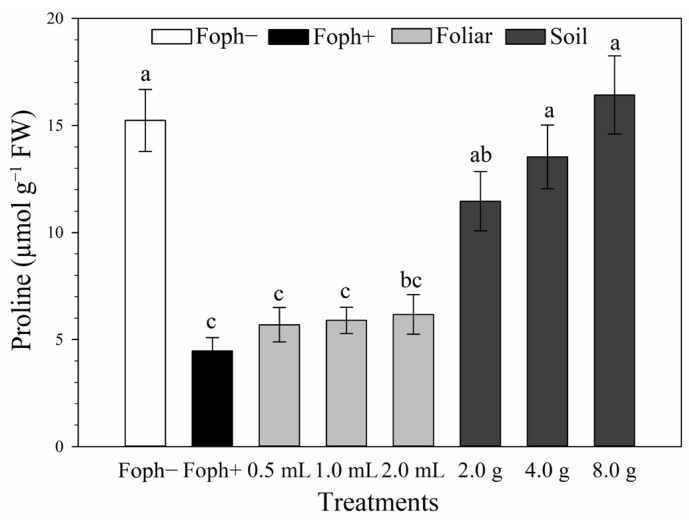
Effect of foliar and soil Si application on vascular wilt in cape gooseberry plants at 70 DAI on proline concentration (µmol g^−1^FW). Bars represent the standard error, and letters indicate statistically significant differences according to Tukey’s test (*p* ≤ 0.05).

Proline content showed significant differences (*p* < 0.0001). The Foph− control had 15.2 µmol g^−1^ FW, similar to soil Si treatments, with the highest value in the 8 g treatment (16.4 µmol g^−1^ FW) (Figure 4).

**Figure 5:**
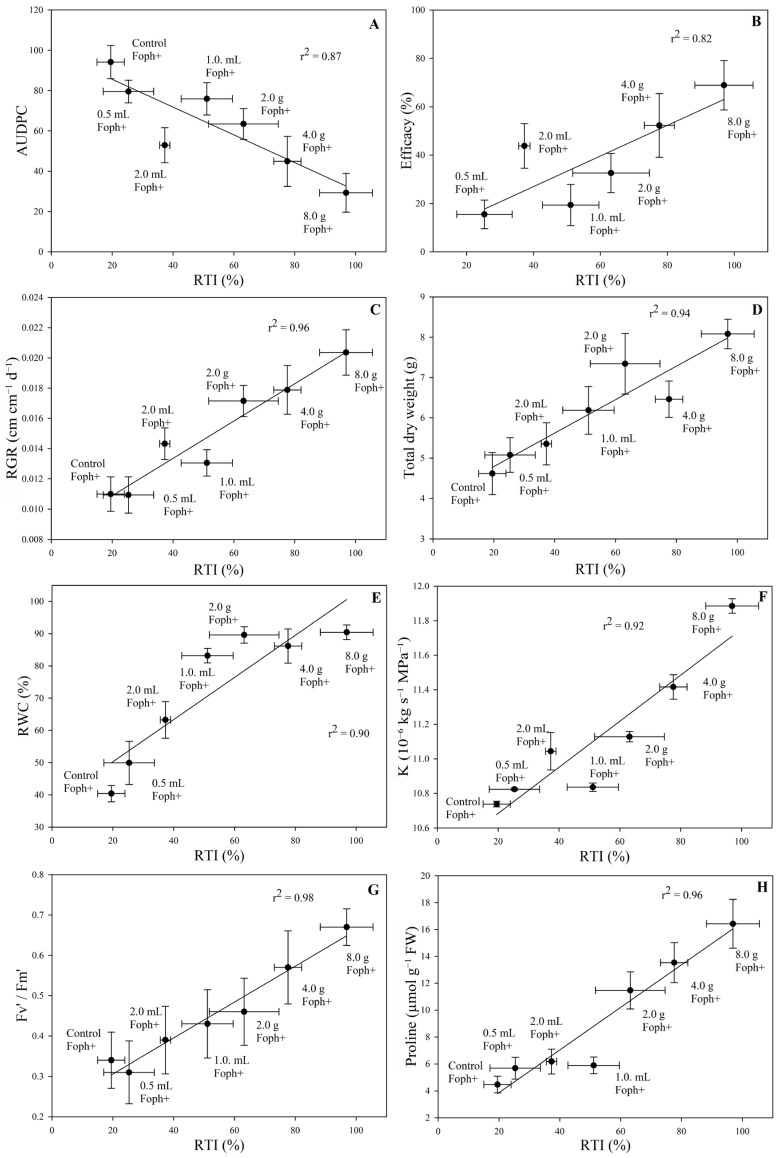
Correlation between (**A**) AUDPC, (**B**) efficacy, (**C**) RGR, (**D**) total dry weight, (**E**) RWC, (**F**) K, (**G**) Fv’/Fm’, (**H**) proline content, and RTI in cape gooseberry plants with Foph and treated with foliar Si (0.5, 1.0, and 2.0 mL) or soil-applied Si (2.0, 4.0, and 8.0 g) at 70 DAI. Each point represents the mean of five plants. Vertical and horizontal bars indicate the standard error per treatment (n = 5).

AUDPC showed a negative correlation with RTI (r^2^ = 0.87; *p* = 0.0105), indicating that soil applications at 4 and 8 g had the lowest disease progression and highest tolerance (Figure 5A). Efficacy, RGR, total dry weight, RWC, K, Fv’/Fm’, and proline content showed strong positive correlations with RTI (r^2^ ranging from 0.82 to 0.98), particularly in the 4 and 8 g soil treatments (Figure 5B–H).

**Figure 6:**
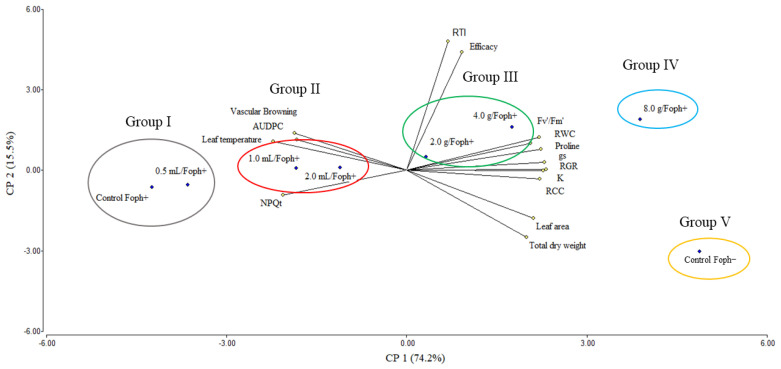
Biplot analysis of cape gooseberry plants (*Physalis peruviana* L.) inoculated with Foph at 70 DAI under foliar and soil Si application. Abbreviations: Foph−, absolute control without Foph inoculation and without Si; Foph+, plants inoculated with Foph without Si; 0.5 mL, plants inoculated with Foph and foliar Si application at 0.5 mL L^−1^; 1.0 mL, plants inoculated with Foph and foliar Si at 1.0 mL L^−1^; 2 mL, plants inoculated with Foph and foliar Si at 2.0 mL L^−1^; 2.0 g, plants inoculated with Foph and soil-applied Si at 2.0 g kg^−1^; 4.0 g, plants inoculated with Foph and soil-applied Si at 4.0 g kg^−1^; 8.0 g, plants inoculated with Foph and soil-applied Si at 8.0 g kg^−1^. PC, principal component.

Physiological and biochemical responses at 70 DAI (Figure 6) explained 90% of the variation observed according to principal component analysis (PCA). The variables are represented by vectors, whereas the Si application method and doses are identified by points. Foph− plants formed a distinct group (group V), while Foph+ plants without Si were in the opposite sector (group I), confirming the negative impact of the pathogen. Differential effects associated with Si applications were observed. The 8 g soil treatment clustered close to Foph− (group IV), while 2 and 4 g soil treatments formed an intermediate group (group III). Foliar treatments clustered near Foph+ (group II), indicating a weaker mitigation effect.

**Figure 7:**
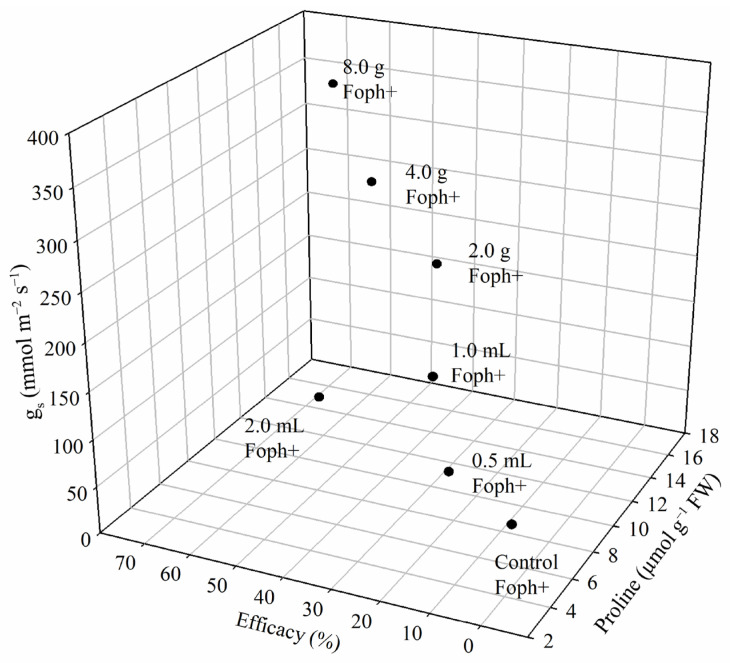
Efficacy percentage, proline content, and stomatal conductance *g_s_*, for cape gooseberry plants inoculated with Foph (Foph+ control), treated with foliar Si applications (0.5, 1.0, and 2.0 mL with Foph+) and soil-applied Si (2.0, 4.0, and 8.0 g with Foph+) at 70 DAI. Data represent the mean of five measurements.

The three-dimensional plot (efficacy percentage, proline content, and *g_s_*) confirmed that soil application of 4.0 and 8.0 g Si improved plant response against vascular wilt caused by Foph (Figure 7).

### 3.3. In Vitro Effect of Silicon on Mycelial Growth of Foph

**Figure A3:**
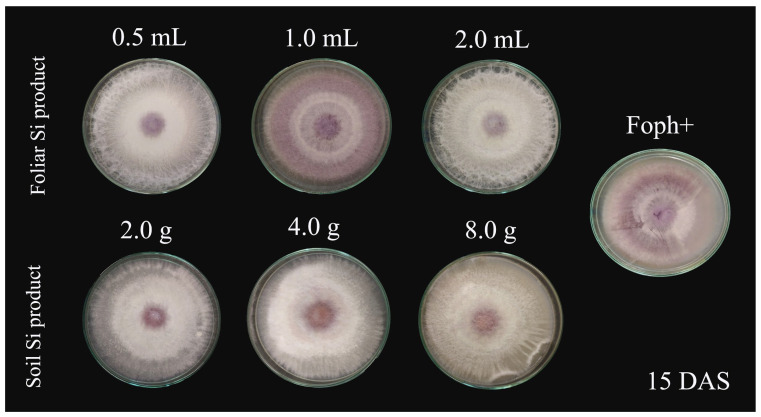
Mycelial growth of Foph MAP5 under the foliar-applied product (MisilK 360) at three doses (0.5, 1.0, and 2.0 mL L^−1^), and under the soil-applied product (Silizeo-K) at three doses (2.0, 4.0, and 8.0 g kg^−1^). Images were taken at 15 DAS.

**Figure A4:**
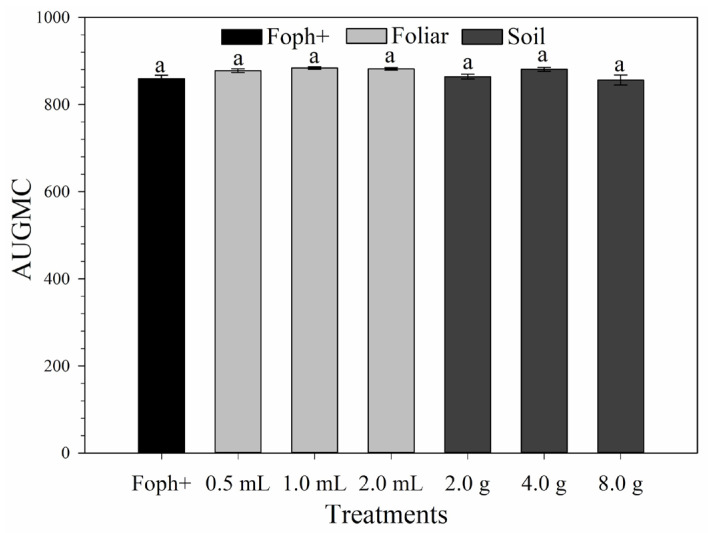
AUGMC of Foph MAP5 under the foliar-applied product (MisilK 360) at three doses (0.5, 1.0, and 2.0 mL L^−1^), and under the soil-applied product (Silizeo-K) at three doses (2.0, 4.0, and 8.0 g kg^−1^). Bars represent the standard error, and letters indicate statistically significant differences according to Tukey’s test (*p* ≤ 0.05).

The soil and foliar applied Si products did not show significant inhibitory effects on the in vitro mycelial growth of Foph at any of the evaluated doses (Appendix A, Figure A3 and Figure A4).

## 4. Discussion

Vascular wilt of cape gooseberry is the most limiting and impactful disease in the main production areas in Colombia, as well as in other Andean regions [58]. Despite its importance, available management strategies remain limited and have relied primarily on fungicide use. However, alternative approaches have been explored [29,32,45,59]. In this context, Si has been proposed as a sustainable alternative capable of inducing tolerance against diverse pathogens, particularly those associated with vascular diseases [8,52]. In this study, the effect of soil and foliar Si applications on the severity of the disease was evaluated. The results demonstrate that Si application in cape gooseberry significantly reduced Foph severity and improved plant performance, thereby confirming the potential of this element as a component of integrated crop management.

Disease analysis showed that Si treatments, particularly soil applications significantly reduced the progression of vascular wilt caused by Foph. Additionally, these treatments exhibited the highest levels of disease control efficacy. These findings are consistent with Sun et al. [52], who observed that soil drench application of Si (1.2 mmol Na_2_SiO_3_·9H_2_O per plant) reduced cucumber wilt caused by *F. oxysporum* f. sp. *cucumerinum* with an efficacy of 69.3%. Similarly, Huang et al. [12] reported a 52.5% reduction in AUDPC following the application of 60 mg Si per plant (0.6 g Na_2_SiO_3_·9H_2_O) in tomato inoculated with *F. oxysporum* f. sp. *radicis-lycopersici*, compared with inoculated control without Si. Likewise, Fortunato et al. [23] observed a 51.9% reduction in AUDPC in banana plants affected by *F. oxysporum* f. sp. *cubense* through the application of 1.75 g calcium silicate (10.5% Si).

The result obtained indicates that, under the Si sources and doses evaluated, the protective effect of Si against vascular wilt in cape gooseberry seems not to be associated with direct antifungal activity but rather with plant-mediated mechanisms. Similar findings were reported by Belhedi et al. [60], who found no significant difference in the mycelial growth of *Fusarium brachygibbosum* after the application of SiO_2_ nanoparticles compared with the control. Although some studies have reported partial inhibition (15–50%) of fungal growth depending on Si concentration and formulation in different *Fusarium* species [52,61,62,63], these responses appear to be context-dependent.

The greater efficacy of Si in mitigating vascular wilt was also reflected in a more favorable plant performance of cape gooseberry plants infected by Foph. Infection by vascular pathogens such as *F. oxysporum* compromises plant growth, water status, and photosynthetic functionality because of xylem obstruction, water imbalance, increased oxidative stress, and the production of fungal toxins, including fusaric acid, dehydrofusaric acid, and lycomarasmin, which contribute to wilt symptom development [56,64,65]. Consistent with this pattern, plants inoculated only with Foph showed marked reductions in RTI, stomatal conductance, relative chlorophyll content, and maximum quantum efficiency of photosystem II, along with increased leaf temperature. In contrast, soil Si applications maintained these variables at levels close to those observed in non-inoculated plants, suggesting effective attenuation of pathogen-induced physiological stress. Similar results have been reported in other pathosystems, where Si contributed to preserving stomatal conductance, photosynthetic efficiency, and plant water status in *Fusarium*-infected plants, thereby enhancing tolerance to biotic stress [52,66,67,68].

Proline content has been widely used as a biochemical marker to evaluate plant responses to stress conditions [69]. In this study, soil applications of Si, increased proline content compared with the Foph-inoculated treatment without Si. This increase showed a strong correlation with the RTI, suggesting an enhanced capacity to cope with Foph-induced biotic stress. Increases in proline concentration and defense enzyme activity following Si application have previously been reported in tomato plants affected by fungal and bacterial pathogens [62]. Similarly, Elshahawy et al. [70] observed comparable responses in onion and garlic under attack by *Stromatinia cepivora*, while Mahdikhani et al. [71] reported increased proline content in squash infected with *F. solani* f. sp. *cucurbitae* after Si application compared with the control without Si.

The reduction in the severity of Foph wilt observed in Si-treated cape gooseberry plants may be associated with the activation of structural, biochemical, and molecular defense mechanisms that restrict colonization and progression of Foph within vascular tissues as has been reported previously [8,72]. Si has been found to accumulate in root and vascular cell walls, where it is deposited as silica or associated with cell wall polymers, promoting lignification and wall thickening. This constitutes a physical barrier that hinders penetration and movement of vascular pathogens [73,74]. In addition, Si application has been associated with increased synthesis of phenolic compounds, phytoalexins, and lignin, as well as activation of antioxidant defense-related enzymes [72,75]. Several studies have demonstrated that Si can modulate the expression of defense-related genes and signaling pathways mediated by phytohormones, which are key pathways in responses to vascular pathogens [8,76,77]. In this context, it can be hypothesized that Si would act as a resistance modulator that enables a more efficient defensive response of the host against Foph infection, as described in other pathosystems [8]. However, future studies must be conducted to reveal the mechanisms involved in the Si-mediated resistance observed in the Foph-*Physalis peruviana* interaction.

The mode of Si application differentially influenced the response of cape gooseberry plants to Foph. Overall, soil application of Si showed greater efficacy in reducing disease severity compared with foliar application, in agreement with reports from other pathosystems, including *Podosphaera xanthii* in *Cucumis sativus* [78], *Colletotrichum gloeosporioides* in *Capsicum annuum* [79], foliar diseases in *Pisum sativum* [26], and *Fusarium* spp. in *Triticum* spp. [80,81]. This greater efficacy may be related to more efficient root absorption of Si and its subsequent translocation through the xylem, favoring its accumulation in vascular tissues and aerial organs, where it can exert a more consistent effect on host defense mechanisms [73,74]. In contrast, foliar application of Si often shows lower persistence and systemic mobility due to surface accumulation and potential wash-off, limiting its redistribution to internal tissues and reducing its effectiveness against vascular pathogens [82]. Moreover, the higher efficacy of soil-applied Si may also be associated with priming effects, as Si supplied to roots can induce the expression of defense-related enzymes [83].

Multivariate analysis allowed the integration of physiological, photosynthetic, and disease severity responses, demonstrating that Si application coordinately modulates tolerance mechanisms in cape gooseberry under vascular wilt stress. The observed association between photosynthetic variables and lower disease severity suggests that Si contributes to maintaining photosynthetic apparatus functionality under Foph stress, possibly through improved water status, membrane stability, and attenuation of oxidative stress, as reported in other vascular pathosystems [8,73,74]. In this context, our results reinforce the hypothesis that Si does not act on a single process but exerts a systemic effect integrating structural, physiological, and metabolic responses. Likewise, the inverse relationship between disease severity index and physiological parameters supports the use of integrative indicators such as the RTI to more realistically describe plant functional status under stress [45,55,59]. Collectively, these results position Si as a key modulator of physiological resistance in cape gooseberry against Foph, integrating disease reduction with maintenance of plant physiological status.

The evidence generated in this study suggests that the effect of Si in mitigating vascular wilt in cape gooseberry cannot be explained solely by a direct reduction in disease severity, but rather by the induction of a more robust and functional physiological state under biotic stress. The convergence of reduced disease progression, preservation of photosynthetic performance, maintenance of plant water status, and increased accumulation of tolerance-associated metabolites such as proline supports the hypothesis that Si acts as a systemic modulator of the plant–pathogen interaction, as proposed in another host–*Fusarium* pathosystems [8,52,74]. These findings expand current knowledge on the role of Si in vascular pathosystems and provide strong evidence for its incorporation as a complementary component within integrated management strategies for vascular wilt in cape gooseberry, particularly in scenarios where chemical control options are limited or unsustainable. Nevertheless, future studies aimed at elucidating the underlying molecular mechanisms, as well as field evaluations under natural pathogen pressure, soil variability and environmental stress, will be essential to reinforce the use of Si as an agronomic tool in this crop.

## 5. Conclusions

Vascular wilt caused by Foph is a limiting disease in cape gooseberry cultivation. The disease can significantly reduce plant development, physiological and photosynthetic status, and ultimately cause plant wilting. In this study, Si application significantly reduced the severity of vascular wilt caused by Foph in cape gooseberry plants, with soil application—particularly at doses of 4 and 8 g per plant—being the most effective (52% and 69%, respectively). Disease mitigation was associated with preservation of physiological and photosynthetic performance in infected plants, as well as with an increase in proline content, indicating an improved capacity to respond to Si-induced biotic stress. Overall, the integration of disease severity, physiological, and photosynthetic variables through multivariate analysis demonstrates that Si acts as a systemic modulator of cape gooseberry tolerance to vascular wilt, beyond an isolated effect on disease progression. These findings support the use of Si as a complementary component within vascular wilt management strategies in cape gooseberry; however, future studies under field conditions and in combination with other management strategies, including crop yield evaluation, will be necessary to consolidate its commercial application. The findings of our study highlight the potential role of Si in Andean fruit production systems, providing valuable insights for sustainable agricultural practices. The significance of this lies in the possibility that Si application could reduce the need for synthetic fungicides making cape gooseberry production more sustainable and better suited for organic or zero-residue markets. Additionally, it expands the range of tools available to growers for integrated disease management, all while minimizing environmental impact.

## Figures and Tables

**Table 1:** Non-destructive physiological parameters of cape gooseberry plants inoculated with *Fusarium oxysporum* f. sp. *physali* (Foph) under different foliar Si treatments (0.5, 1.0, and 2.0 mL L^−1^) and soil Si treatments (2.0, 4.0, and 8.0 g kg^−1^) at 63 and 70 DAI. RGR, leaf temperature, and stomatal conductance (*g_s_*). Control without Foph and without Si (Foph−) and control with Foph without Si (Foph+).

Treatment	Relative Growth Rate (RGR) (cm cm^−1^ d^−1^)	Leaf Temperature (°C)		Stomatal Conductance (*g_s_*) (mmol CO_2_ m^−2^ s^−1^)	
	DAI	DAI		DAI	
	70	63	70	63	70
Foph− absolute control	0.0217 a	22.5 c	19.6 d	307.2 a	352.0 a
Foph+	0.0110 c	27.6 ab	23.8 ab	57.6 c	68.6 d
0.5 mL/Foph+	0.0110 c	27.8 a	26.0 a	102.1 c	89.2 d
1.0 mL/Foph+	0.0130 bc	26.9 ab	23.3 abc	186.3 abc	179.9 bcd
2.0 mL/Foph+	0.0144 bc	24.3 abc	22.1 bcd	142.5 bc	131.2 cd
2.0 g/Foph+	0.0172 ab	26.5 abc	24.7 ab	184.9 abc	222.3 bc
4.0 g/Foph+	0.0180 ab	26.7 abc	23.4 ab	193.6 abc	273.1 ab
8.0 g/Foph+	0.0204 a	23.3 bc	20.6 cd	269.1 ab	341.1 a
Significance (*p* value) ^1^	<0.0001	0.0012	<0.0001	<0.0001	<0.0001
CV (%) ^2^	28.6	10.6	10.2	54.4	55.7

^1^ Values within columns followed by different letters are significantly different according to Tukey’s test (*p* ≤ 0.05). ^2^ CV: coefficient of variation (*n* = 5).

**Table 2:** Photosynthetic parameters of cape gooseberry plants inoculated with *Fusarium oxysporum* f. sp. *physali* (Foph) under different foliar Si treatments (0.5, 1.0, and 2.0 mL L^−1^) and soil Si treatments (2.0, 4.0, and 8.0 g kg^−1^) at 63 and 70 DAI. RCC (SPAD), Fv’/Fm’, φII, LEF, qP, NPQt, φNO, and φNPQ. Control without Foph and without Si (Foph−) and control with Foph without Si (Foph+).

Treatment	Relative Chlorophyll Content (SPAD Units)		Maximum Quantum Efficiency of PSII (*F_v’_*/*F_m’_*)		Quantum Efficiency of PSII Under Light Conditions (ϕ_II_)		Linear Electron Flow (LEF)		Photochemical Quenching (qP)		Non-Photochemical Quenching (NPQt)		Uncontrolled Nonphotochemical Dissipation (ϕ_NO_)		Controlled Non-Photochemical Dissipation (ϕ_NPQ_)	
	DAI		DAI		DAI		DAI		DAI		DAI		DAI		DAI	
	63	70	63	70	63	70	63	70	63	70	63	70	63	70	63	70
Foph– absolute control	48.2 a	48.2 a	0.63 a	0.59 ab	0.52 a	0.48	61.1 ab	79.7	0.61	0.68	1.9 a	2.6	0.18 a	0.15	0.31 a	0.36
Foph+	31.2 c	27.2 b	0.45 a	0.34 ab	0.38 a	0.31	38.5 b	72.6	0.65	0.71	4.3 a	8.0	0.12 a	0.09	0.50 a	0.60
0.5 mL/Foph+	35.1 abc	32.7 ab	0.54 a	0.31 b	0.47 a	0.28	41.9 b	92.4	0.59	0.70	2.3 a	11.3	0.17 a	0.08	0.36 a	0.64
1.0 mL/Foph+	32.6 bc	31.0 ab	0.45 a	0.43 ab	0.34 a	0.35	32.0 b	76.4	0.64	0.66	5.1 a	7.3	0.11 a	0.11	0.55 a	0.54
2.0 mL/Foph+	33.6 abc	32.8 ab	0.47 a	0.39 ab	0.37 a	0.33	38.7 b	100.9	0.67	0.81	5.0 a	8.8	0.12 a	0.09	0.51 a	0.57
2.0 g/Foph+	39.4 abc	41.0 ab	0.53 a	0.46 ab	0.47 a	0.37	51.7 b	104.0	0.62	0.67	2.8 a	7.0	0.15 a	0.12	0.38 a	0.51
4.0 g/Foph+	46.4 abc	41.5 ab	0.62 a	0.57 ab	0.48 a	0.51	96.1 a	110.2	0.65	0.59	2.6 a	2.1	0.15 a	0.19	0.37 a	0.30
8.0 g/Foph+	46.8 ab	43.4 ab	0.67 a	0.67 a	0.51 a	0.51	60.7 ab	81.4	0.65	0.63	2.3 a	2.1	0.16 a	0.17	0.33 a	0.33
Significance (*p* value) ^1^	0.0018	0.0298	0.0210	0.0177	0.0267	0.1693 NS	0.0011	0.9504 NS	0.3432 NS	0.0670 NS	0.0307	0.1113 NS	0.0275	0.0542 NS	0.0243	0.1249 NS
CV (%) ^2^	24.5	30.7	24.5	41.2	27.2	41.3	59.6	55.1	9.2	14.2	74.3	93.0	32.1	48.8	39.3	45.4

^1^ Values within columns followed by the same letters are not significantly different according to Tukey’s test (*p* ≤ 0.05). ^2^ CV: coefficient of variation. NS, not significant. (n = 5).

## Data Availability

The original contributions presented in this study are included in the article. Further inqueries can be directed to the corresponding authors.

## Abbreviations

The following abbreviations are used in this manuscript:

AUDPC	Area under the disease progress curve
AUGMC	Area under the mycelial growth curve
CP	Principal component
DAI	Days after inoculation
DAS	Days after sowing
Foph	*Fusarium oxysporum* f. sp. *physali*
Foph−	Non-inoculated control without pathogen
Foph+	Inoculated control with pathogen
*g_s_*	Stomatal conductance
K	Plant hydraulic conductance
LEF	Linear electron flow
NPQt	Non-photochemical quenching
PCA	Principal component analysis
qP	Photochemical quenching
RGR	Relative growth rate
RTI	Relative tolerance index
RWC	Relative water content
Si	Silicon
Fv’/Fm’	Maximum quantum efficiency of photosystem II
PDA	Potato dextrose agar
WC	Water content
φII	Quantum efficiency of PSII under light conditions
φNO	Uncontrolled nonphotochemical dissipation
φNPQ	Controlled non-photochemical dissipation
